# Role of Machine Learning in Resource Allocation Strategy over Vehicular Networks: A Survey

**DOI:** 10.3390/s21196542

**Published:** 2021-09-30

**Authors:** Ida Nurcahyani, Jeong Woo Lee

**Affiliations:** 1 School of Electrical and Electronics Engineering, Chung-Ang University, Seoul 06974, Korea; idanurcahyani@cau.ac.kr; 2 Department of Electrical Engineering, Universitas Islam Indonesia, Yogyakarta 55584, Indonesia

**Keywords:** vehicular network, resource allocation, machine learning, survey paper

## Abstract

The increasing demand for smart vehicles with many sensing capabilities will escalate data traffic in vehicular networks. Meanwhile, available network resources are limited. The emergence of AI implementation in vehicular network resource allocation opens the opportunity to improve resource utilization to provide more reliable services. Accordingly, many resource allocation schemes with various machine learning algorithms have been proposed to dynamically manage and allocate network resources. This survey paper presents how machine learning is leveraged in the vehicular network resource allocation strategy. We focus our study on determining its role in the mechanism. First, we provide an analysis of how authors designed their scenarios to orchestrate the resource allocation strategy. Secondly, we classify the mechanisms based on the parameters they chose when designing the algorithms. Finally, we analyze the challenges in designing a resource allocation strategy in vehicular networks using machine learning. Therefore, a thorough understanding of how machine learning algorithms are utilized to offer a dynamic resource allocation in vehicular networks is provided in this study.

## 1. Introduction

The vehicular network is the main component in smart mobility and is the main source of information and communication technology (ICT) in smart cities [1]. The concept begins with the vehicular ad hoc network (VANET) which is a part of the ad hoc network. The vehicular network has a decentralized nature with a dynamic topology where nodes can join and separate themselves independently and can be built without the need for existing infrastructure. A vehicle can directly communicate with other vehicles (vehicle-to-vehicle—V2V) at a certain distance through an onboard unit (OBU) that has sensing and communication capabilities via a wireless network. In addition, a vehicle can also communicate with the infrastructure (vehicle-to-infrastructure—V2I) to obtain information regarding traffic conditions and infrastructure. The evolution of V2I towards vehicle-to-everything (V2X) communication then makes the vehicular network a part of the intelligent transport system (ITS) which can support smart mobility in which vehicles can communicate with the surrounding environment [2].

According to [3], the Global Internet of Cars in 2020 reached USD 115.7 billion and is predicted to increase more than six times by 2027. This shows the high need for smart vehicles that can provide safer, more comfortable, environmentally friendly transportation, and provide entertainment during transportation. This smart vehicle continuously senses, collects, and transmits data so that it will affect the volume of data in the vehicular network. Applications on the vehicular network include active safety applications, non-safety applications, and infotainment [4]. Each type of application has different quality of service (QoS) requirements.

Various types of applications, media, and communication technologies involved in V2X will require a mechanism for managing and assigning network resources so that the processing and exchanging of information can run properly. Moreover, vehicular network communication uses wireless media that are very susceptible to interference, attenuation, fading, and dispersion. In addition, the vehicular network has a dynamic topology that is influenced by the movement of nodes so that the data transmission process must be executed during a very short period. Due to these characteristics, the role of a dynamic resource allocation mechanism that can quickly adjust its allocation policy according to network conditions is needed so that network resources can be efficiently utilized. Moreover, fulfilling the QoS of each application also requires the proper assignment of resources for each data transmission to guarantee successful transmissions.

The problem of resource allocation in vehicular networks has attracted the interest of many researchers. There are various types of network resources in V2X, including time slots, channels, computing abilities and power levels [5]. Network resources must be arranged so that they are optimally distributed to all active users in the network. Researchers used various techniques in managing and allocating these resources, ranging from conventional optimization techniques to implementing artificial intelligence (AI) algorithms. Due to the characteristics of high mobility and topology change, a dynamic resource allocation mechanism is preferred to handle vehicular networks [6].

Various data collected and transmitted on the vehicular network can be categorized as big data [7]. Moreover, vehicular nodes are seen as not only exchanging information but also performing data gathering, computing, and storing [8]. The characteristics of big data, namely volume, variety, velocity, value, and veracity (5V) [9] can also be applied to vehicular networks so that the problems that arise can be solved with big data techniques such as AI [8]. The implementation of AI in the field of telecommunication has provided opportunities for the development of intelligent networks that can autonomously assist decision making based on local observations.

Researchers have been using various considerations and strategies in implementing AI algorithms for resource allocation in vehicular networks. The development of AI implementation in vehicular networks shows the potential of this technique to manage and allocate network resources. This paper investigated how AI algorithms were utilized in vehicular resource allocation mechanisms. Through our literature study, several survey papers with similar themes [6,10,11,12,13] were found. In detail, the overall contribution of these similar survey papers can be seen in Table 1. However, after perusing the papers, we found that a specific discussion on the role of AI algorithm implementation for a vehicular network resource allocation mechanism has never been performed before. The contributions of this paper include:

1. A discussion of several machine learning scenarios used by previous researchers in managing and allocating network resources;
2. Classifying machine learning roles and strategies in resource allocation strategies for each machine learning category;
3. Identifying the challenges in implementing machine learning algorithms for vehicular network resource allocation.

The rest of the paper is written in the following order. An introduction to vehicular networks, especially high-mobility radio access technologies (RATs) and mobile big data in V2X is discussed in Section 2. Then, a brief introduction to the various AI algorithms that have been implemented for resource management and allocation in V2X is given in Section 3. Section 4 presents a detailed discussion of the strategies that have been carried out by previous researchers when allocating network resources in V2X. Discussion of the challenges and opportunities of implementing AI algorithms for V2X resource allocation is shown in Section 5, and then followed by conclusions from this survey paper in Section 6.

## 2. Vehicular Network Preliminary

**Figure 1:**
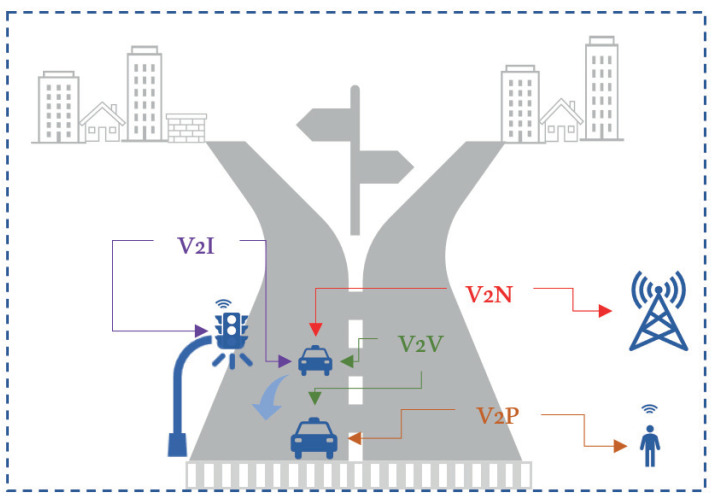
V2X communication scenarios.

In V2X communication, a vehicle can directly communicate with other vehicles (V2V), roadside infrastructure (V2I), pedestrians (V2P), and with the network (V2N) as a device. Figure 1 depicts the V2X communication schemes. A vehicle has communication and computation devices embedded in its OBU, similar to the “things” in the Internet of Things (IoT). Nevertheless, due to the challenging environment of vehicular networks, characterized by their high mobility and dynamic topology, the RATs used in device-to-device (D2D) communication encounter many challenges when applied to the vehicular network.

### 2.1. Direct Communication Technology for High Mobility

With the help of industry and academia, several standardization institutions have been competing to standardize RATs that can support vehicular communication. Some examples are the IEEE 802.11p-based RAT, which is adopted by Direct Short-Range Communication (DSRC) from the USA, and ITS-G5 from European Telecommunications Standards Institute (ETSI) [14], Cellular-V2X (C-V2X), which is a standard from the 3rd Generation Partnership Project (3GPP), Wi-Fi, White-Fi, Bluetooth, WiMax, infrared, and visible light communication (VLC) [15]. However, some of these RATs are considered unable to satisfy vehicular application communication requirements, so new RATs continue to be pursued to support autonomous vehicle communication in the future [5,16]. This is due to the characteristics of the vehicular network, which has high mobility with many nodes that are spread out and use various protocols for communication. The potential solution to enable direct communication between vehicles in a challenging environment has been brought by DSRC-WAVE and C-V2X access technologies.

Different standardization bodies work on developing DSRC V2X standards. In the USA, DSRC is standardized by IEEE as a wireless access in vehicular environment (WAVE). In Europe, the DSRC technology is referred to as ITS-G5 and is standardized as ETSI EN 302 663 [17]. On the other hand, C-V2X uses a cellular network based on the 3GPP standard. The DSRC physical layer uses orthogonal frequency division multiplexing (OFDM) combined with convolutional code. The DSRC radio spectrum is allocated in a band of 5.9 GHz. This radio spectrum is especially dedicated to DSRC applications. IEEE 802.11p is used as the standard for physical and data link layers for DSRC-WAVE and ITS-G5. The system architecture, set of services, and interfaces are defined by WAVE [18]. Through WAVE, data transfer can be performed in non-IP-based via WAVE Short Message Protocol (WSMP) or IP-based [19] scenarios. Since IEEE 802.11p is based on an ad hoc network, problems such as hidden terminals and network congestion caused by the accumulation of vehicles in an area can occur. Therefore, 3GPPP built a special communication protocol that can be implemented in ITS. With the help of pre-existing LTE evolved-NodeB (eNB), C-V2X offers a wider coverage area with large capacity and low latency [15].

LTE-V2X, better known as C-V2X, is a standard communication protocol under 3GPP which has a flat all-IP infrastructure. C-V2X also uses a frequency band between 5.855 and 5.925 GHz in band 47. The C-V2X platform was developed from LTE-D2D, which is the 3GPP Release 12 (Rel-12) standard. It was later refined to Rel-13 and beyond for public safety communications. With a packet switch-based architecture, it can reduce logical network nodes to lessen infrastructure costs and minimize latency on the network. However, direct communication on V2X, which can provide ultra-high reliability and very low latency, is still unable to be fulfilled by LTE cellular-based radio technology [20]. Hence, a new RAT that can meet the service requirements for this V2X application is needed.

5G technology, which is a new global wireless standard from 3GPP, is associated as an answer to the challenges of massive device communication in the future. It is described as the key technology to enable connected and cooperated autonomous driving [21]. The implementation of a vehicular network through 5G technology has a lot of potential, not only in terms of increasing capacity and data rate but also for supporting coverage and device mobility. In addition, the design of a 5G infrastructure is based on softwarization and virtualization so that the process of deploying, scaling, and managing networks becomes easier [22]. This makes the 5G network very suitable for D2D communication in the Internet of Vehicles (IoV) and allows the wider implementation of advanced technologies/tools such as the vehicular Cloud, fog computing, and network slicing to support its performance targets [5].

### 2.2. Intelligent Vehicular Network with Machine Learning

**Figure 2:**
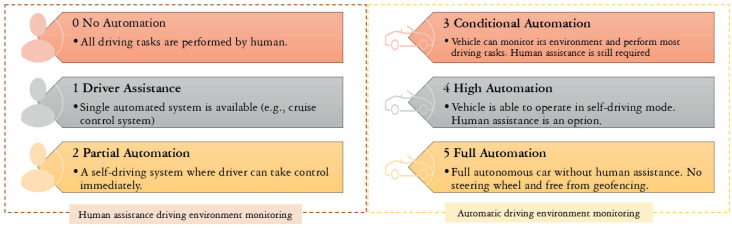
Autonomous vehicle levels by SAE.

The intelligent vehicles and transport system for the future will cause a surge in the amount of data on the vehicular network. The Society of Automotive Engineers (SAE) stated that there are six levels of vehicles from an autonomous point of view [23], as shown in Figure 2. The number of vehicles with automated system monitors, i.e., level 3 and above, is expected to reach eight million vehicles by 2025 [24]. The higher the level of autonomy is, then the more the vehicle relies on sensors to replace human interaction in driving activities. The involvement of cameras, RADAR, LiDAR, GPS, and various sensors in the body of smart vehicles will make a vehicle capable of generating gigabytes of data per second. This exponential escalation of data generated, transmitted, and collected by vehicular networks introduces the paradigm of mobile big data (MBD) in the vehicular network [8,25].

MBD include all the data collected, managed, and processed by a device or a tool at a certain time. These diverse data with a massive size certainly need to be systematically analyzed to retrieve useful information for road users. Smart vehicles are now equipped with computing and storage devices that can be used to process these data to improve comfort and safe driving. However, processing large and diverse data requires machines with high computing capabilities.

Some of the challenges for MBD in V2X include (a) a large number of sensors involved in assisting the environmental monitoring process so that data duplication will be massive; (b) the limitations of processing and storage units on OBUs owned by vehicles; and (c) the vehicle environment that changes very quickly due to the vehicle’s mobility. In addition, road safety applications, as well as traffic efficiency and management applications, must provide real-time information; thus, requiring immediate processing and transmission. To overcome these challenges, new technology and paradigms revolutionizing conventional vehicular networks to become intelligent vehicular networks are needed to support the use of MBD to improve service and customer satisfaction.

The explosion of machine-type communication, the evolution of technology, and the increasing demand for data in the vehicular network make the telecommunication and automotive industries transform the infrastructure to introduce new network models and service capabilities. 5G technology comes with an architecture based on a software defined network (SDN) and network function virtualization (NFV) designed to support massive machine types of communication (mMTC). Softwarization, virtualization, and machine learning concepts are also introduced to support vehicular communication. These concepts can be employed to enable mobile big data in V2X [26].

Softwarization through SDN offers flexibility, programmability, and centralized control so that network management and development processes can easily be carried out. Virtualization via NFV as well as Cloud and edge computing enable the process of offloading computational loads from OBUs to minimize computational delay during data processing. Meanwhile, machine learning provides the ability for the network to process such a large amount of data so that valuable information can be retrieved and used for the benefit of road users’ safety and traffic efficiency.

These communication technologies and distributed computing systems have become the key to implementing machine learning to respond to the increase in data on telecommunication networks. Machine learning has been projected to be the primary solution for optimizing telecommunication networks with various network types, applications, and service requirements. Through machine learning, the system can directly take solutions without any predefined rules [27]. In addition, applying machine learning techniques to telecommunication networks, especially vehicular networks, can improve network efficiency and adaptability. Moreover, 6G technology—as the evolution of wireless networks in the future—will require ubiquitous intelligence so that it can offer connected intelligence from the core network to end devices [28].

### 2.3. Resource Allocation in Vehicular Network

In DSRC, when a vehicle wants to send a message to another neighboring vehicle, the message is passed to the medium access control (MAC) layer. This layer is responsible for guaranteeing the data delivery process. It decides when a node can transmit, receive, or be silent in the process. The DSRC MAC layer is based on IEEE 802.11p standard, which uses the enhanced distributed coordination function (EDCF) mechanism which employs the carrier sense multiple access with collision avoidance (CSMA/CA) [29]. The CSMA/CA implements the stop-and-wait mechanism to prevent packet collision in the receiver. The message, for example, a safety-related message, is only sent within its lifetime and discarded if its lifetime expires [30]. Using EDCF, IEEE 802.11p can give QoS support to the DSCR applications by forming traffic in different access categories (ACs) based on its priority levels [31].

**Figure 3:**
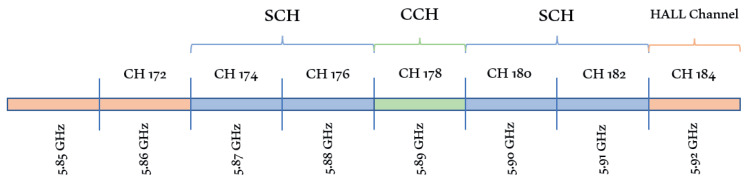
DSRC channel.

DSRC uses the OFDM system to provide up to 1000 m direct communication in V2V and V2I. It operates in a 10 MHz channel that allows data transmission with 3–27 Mbps rates. WAVE has two channel types: service channel (SCH) and control channel (CCH). Each channel has a 10 MHz bandwidth. These channels can be utilized for safety and non-safety applications. V2I direct communication is performed through CCH. This CCH is used to send WAVE short message service (WSMP), which contains application priority, node distance, and minimum rate needed by the application. SCH is employed as an interaction channel between applications that are involved in communication procedures. Non-safety applications’ communication also takes place in SCH. High availability-low latency (HALL) in channel 184 will be used for future needs. Three options of channel access in DSRC for a higher layer to exchange data include continuous access; access alternating between two channels; and immediate channel access [19]. Figure 3 shows the DSRC spectrum for safety and non-safety applications.

C-V2X uses LTE RAT, where the radio resource management (RRM) in C-V2X employs several techniques and procedures. C-V2X communication can be performed by direct link or cellular link. There are three resource allocation modes in reusing a licensed spectrum, which are underlay, overlay, and cellular modes [32]. Underlay and overlay modes are used in direct communication, while the cellular mode is used when the eNB is utilized as an intermediate relay, which is similar to conventional cellular communication.

**Figure 4:**
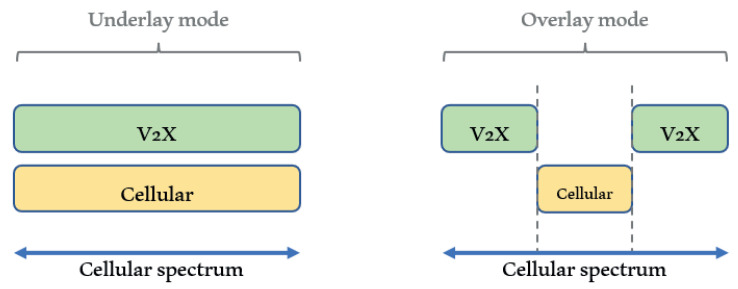
C-V2X mode selection.

In overlay mode, dedicated spectra are allocated for direct communication C-V2X users. The interference problem can be avoided in this mode. Nevertheless, the dedicated spectrum efficiency becomes a challenging problem in this mode. In contrast, C-V2X users share the same available spectra with cellular users in underlay mode. This mode can achieve spectrum efficiency, though C-V2X and cellular users’ interference should be well controlled. Underlay mode is the suggested RRM mechanism in the early-proposed device-to-device (D2D) direct communication. In underlay mode, resource block (RB) can be shared among users with three sharing processes, which are user pairing, user grouping, and user geographic location [33]. C-V2X channel mode selection is shown in Figure 4.

C-V2X time and frequency resources are similar to the LTE structure. The duration of one LTE frame is 10 ms. It is divided into ten smaller sub-frames with 1 ms duration each. This sub-frame has two time slots. Each time slot duration is 0.5 ms and consists of seven OFDM symbols with an extended cyclic prefix. In the frequency domain, the RB size is 180 kHz with 0.5 ms duration. The scheduling process in LTE is performed by the RRM entity that allocates RB in every transmission time interval (TTI).

Many types of research have been done to implement machine learning techniques in optimizing communication networks [34]. Its implementation in vehicular networks with dynamic environments such as channel conditions, network topology, and traffic shapes can especially affect system performance [35]. V2X resource allocation is one of the optimization themes that has attracted the interest of many researchers because of the limited nature of network resources. Seeing the increasing number of smart vehicles and the increasing amount of data involved in vehicular network communication, it is necessary to have a mechanism for regulating the use of dynamic network resources so that network resources can be efficiently used.

The conventional resource types in a communication network can be in the shape of network channels, time slots, and power levels. However, the distributed computing system in the V2X network has introduced other resource types that can be shared among network users, namely computation, storage, and caching resources. Using a distributed computing system, the virtual resource allocation concept is initiated in a vehicular network environment [35]. The unevenly distributed network resource is one of the notable challenges to deliver an efficient networking environment. Most traditional resource allocation algorithms were based on the static environment without considering the dynamic environment of user mobility. This also depends on the use of mathematical formulas that are often non-convex and NP-hard. This is especially the case when adopting a vehicular network perspective where vehicles move with high mobility, resulting in only a brief period during which the allocation strategy can be validly implemented. Moreover, this dynamic environment frequently requires re-executions of the algorithm, which will lead to additional latency in the transmission process [34].

Implementing machine learning to solve resource allocation problems opens a wide range of improvements. Machine learning can dynamically adjust its allocation strategy according to the system’s state environment. It can investigate the relation between parameters that are used in decision-making to make the best policy for this optimization problem. Furthermore, with the increase in the number of devices connected and applications involved, machine learning can learn the dynamic environment and extract some valuable features to benefit many task objectives involved [35].

## 3. Machine Learning Preliminary

The implementation of machine learning in various technologies and applications is now inseparable from human life. Machine learning or learners can convert data into a special algorithm that suits the system’s needs [36]. It is a program that is used for data learning. To efficiently extract information, the type of algorithm and task it performs must be known to match what we want to obtain from the data we have. Although there are various types of machine learning algorithms with several categories, machine learning can generally be classified based on the involvement of human supervision in the learning process. These categories are supervised, unsupervised, and reinforcement learning. In addition, the emergence of deep learning (DL) gives machine learning the ability to solve complicated optimization problems. The following briefly describes each machine learning category. We also provide some references for readers who need a deeper understanding of each subsection.

### 3.1. Supervised Learning

The purpose of supervised learning is to estimate the mapping from the input data. Supervised learning uses the target data as a supervisor in the learning process. The target data constitute a dataset with labels. The supervisor can provide information if the machine makes an error during the learning process with this labeling. This way, the algorithm performs tuning to increase its precision. Based on the type of learning process output, supervised learning has two classes of tasks, namely classification and regression.

In a scenario where a labeled dataset is difficult to obtain, the learning process can be carried out with the involvement of unlabeled data to aid the classification process. This learning process is called semi-supervised learning. Semi-supervised learning is the combination of supervised and unsupervised learning. Generally, this type of learning aims to improve the performance of classification or clustering [37]. This learning method involves a small number of labeled datasets and a large number of unlabeled datasets. Semi-supervised learning enhances clustering tasks by adding supervision information from labeled datasets to guide which unlabeled datasets belong to the same class. Readers can find further study on semi-supervised learning in [38].

Conventional machine learning algorithms process data in batches or chunks [39]. This means that a new batch of data requires a machine learning algorithm to train it from scratch to build the model. With the increase in data generated by machines and users, a new method to quickly and efficiently learn from the data is needed. Stream learning, online learning, and incremental learning are the types of machine learning that can update their models for a given continuous data stream without performing multiple passes over data [40]. Stream learning is closely related to semi-supervised learning [41]. By implementing stream learning, real-time data analytics can be performed. A deeper understanding of stream learning can be found in [39,42,43].

#### 3.1.1. Support Vector Machine

The support vector machine (SVM) is a popular model in the supervised learning class that can be used for various purposes—such as linear and non-linear classification as well as regression and outlier detection [44]. The SVM algorithm is suitable for datasets with a large number of variables but a small sample size. In SVM, the data to be classified will be separated by a line with the equation y=wx+b. Where *x* is the vector point, *w* is the weight which represents the orientation of the hyperplane, and *b* is the hyperplane’s position to the *d*-dimensional space. The decision hyperplane (wx+b=0) in SVM can separate the sample space into two subspaces with a maximum margin. An *n*-dimensional feature space can be separated by a hyperplane with the dimension of n−1.

**Figure 5:**
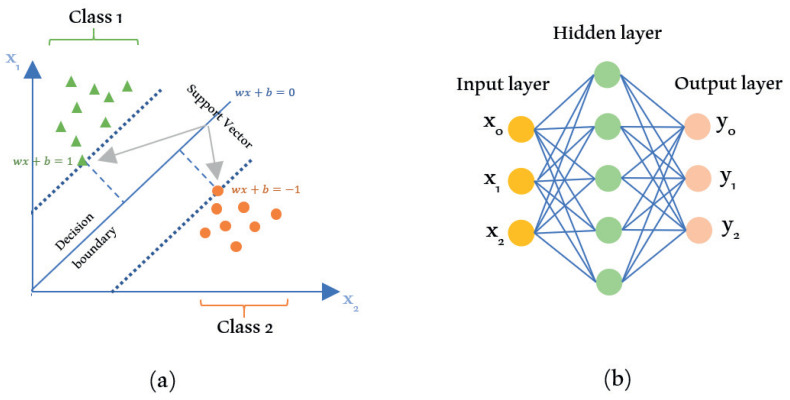
Supervised learning algorithms: (**a**) SVM; (**b**) ANN.

The optimal hyperplane or maximum margin in SVM is the hyperplane that has the maximum distance from its nearest points. The nearest point or sample located in the margin area is called the support vector. The margin domain has two areas, namely the area above the decision hyperplane bounded by a positive hyperplane (wx+b=1) and the area below the decision hyperplane bounded by a negative hyperplane (wx+b=−1). The concept of SVM is shown in Figure 5a.

#### 3.1.2. Artificial Neural Network

This algorithm uses the analogy of the performance of a network of neurons in a biological system. Mathematical artificial neural network (ANN) models mimic the biological structure of the human brain. This way, the ANN algorithm can perform abstraction and generalization, which are special abilities of an organism. The ANN algorithm performs a learning process to recognize patterns from the input data and predict the output of a new similar dataset. Two essential components of ANN are neurons/nodes and synapses/edges. ANN is composed of several layers, which are the input, output, and hidden layers.

The input layer directly interacts with the input data, while the output layer is in charge of predicting the result of the learning process. The hidden layer is the core of ANN, where the computational and learning processes occur. Each layer contains neurons. The neurons of a layer are connected to the next layer’s neurons using edges with a certain weight. The weights on the edges contain information from the input that can play a role in generating or inhibiting the signal that is communicated at each layer. ANN is the basis of the DL algorithm or deep neural network (DNN). DL is one of the subdomains in machine learning that can recognize hidden patterns in the dataset and make predictions from the input data. DL has input and output layers and more than one hidden layer between them that are interconnected. The ANN diagram is shown in Figure 5b. An in-depth discussion of ANN and DL algorithms can be found in [45].

### 3.2. Unsupervised Learning

Datasets in unsupervised learning do not have labels. The purpose of this type of learning is to find specific information from the input data. From this specific information, the task of unsupervised learning will depend on the task of the algorithm. Unsupervised learning is widely used for clustering, namely looking for similar features and creating groups for the data. In addition to clustering, unsupervised learning can also be used for dimension reduction, anomaly detection, and density estimation. Dimension reduction is widely used to reduce computational time if the number of data involved is massive.

Clustering tasks are divided into two categories: namely hard clustering and soft clustering. Hard clustering, such as the *K*-means algorithm, groups data points into only one cluster that has the closest distance to its centroid. The disadvantage of this method is that there is no calculation of the probability that the data points are associated with other clusters. Meanwhile, soft clustering can calculate all the probabilities of a data point associated with all clusters and take the largest value as a cluster of data points. We refer readers who are interested in clustering implementation on VANET to [46].

### 3.3. Reinforcement Learning

**Figure 6:**
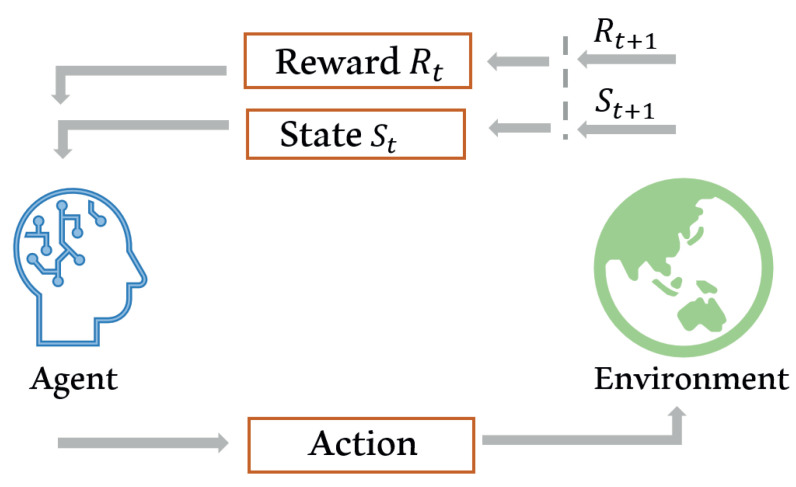
Reinforcement learning model diagram.

In the nature of learning, learners interact with their environment to gain information about cause and effect, the consequences of actions, as well as what is required to achieve certain goals [47]. Reinforcement learning (RL) can only be used when there is no dataset. Instead of a dataset, an environment is provided in the learning system. The optimal decision in RL can be obtained after some period of training. There are two major entities in RL, which are agents and the environment. These two entities, which communicate using three channels which are actions, rewards, and observations. An agent tries to maximize the reward value accumulated during a period in its sequence of actions. Figure 6 shows the common diagram for RL systems. This algorithm is widely used in resource management and allocation due to its decision-making characteristic. Reference [47] provides a detailed explanation about RL theory.

#### 3.3.1. Markov Decision Process

The Markov decision process (MDP) is the basic framework for RL. MDP is an algorithm with a discrete-time state-transition system. MDP has four components in its learning process, namely states *S*, actions *A*, transition model probabilities $P_{r}\left(s_{t+1}|s_{t},a_{t}\right)$, and reward utility *R*. The transition model in MDP is a next-state function that describes the dynamics of the algorithmic learning process. This process uses the Markov property in which the next-state $s_{t+1}$ is affected by the current state $s_{t}$ and the current action $a_{t}$.

The purpose of MDP is to find a suitable policy so that the cumulative reward from the agent is of high value. A policy is the process of mapping from states to actions to show the learner how to take specific action for a set of states it has. In other words, the choice of the learner’s actions depends on the current state and is not influenced by the previous states. This algorithm is the basis of Q-learning, which allows learners to learn independently and make adjustments during the learning process to achieve their goals.

#### 3.3.2. Q-Learning

Q-Learning (QL) is Markovian, where the learning process is carried out to obtain the best policy for MDP. QL is an off-policy value-based learning algorithm. An agent in QL tries to collect the maximum reward through a series of actions in a dynamic environment. For a learning process with a specific purpose, an agent observes the environment it has, then takes action according to its strategy. The agent will receive a reward or punishment in accordance with the actions that have been carried out and take this experience to formulate a new strategy for the following action. This step is repeated as many times as possible until the agent has the optimal strategy and the maximum reward value. The combination of QL and DL produces deep Q-learning (DQL), an advanced version of QL. In DQL, the Q-table in QL is replaced by layers of the neural network so that the algorithm’s stability can be improved.

### 3.4. Deep Learning

**Figure 7:**
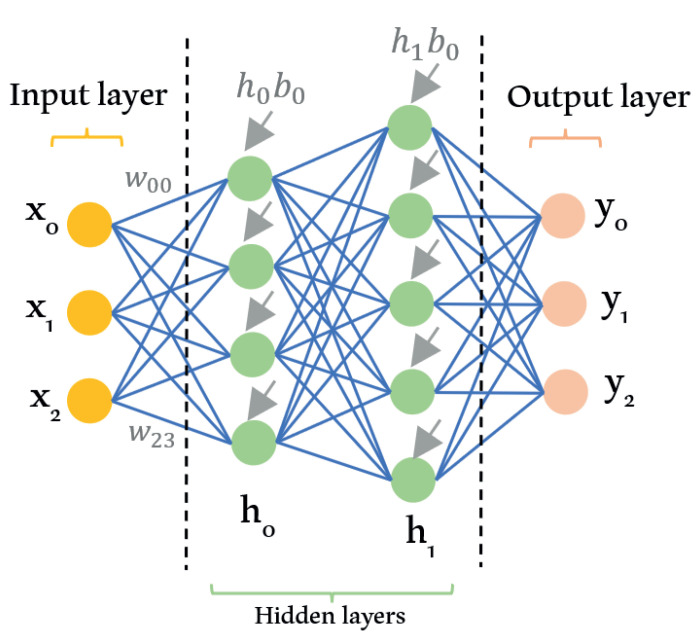
Deep learning model diagram.

While machine learning algorithms enable machines to think with less human intervention, DL emerges as the evolution of machine learning that gives machines the ability to think accurately with a structured model similar to the human brain. DL or DNN is a subdomain of machine learning which can recognize hidden patterns in the dataset and predict an output *Y* based on a given input *X*. The association between inputs and outputs is obtained by utilizing hidden layers constructed from many layers of neural networks. Using this hierarchical architecture, the DL algorithm can predict the expected output with minimum loss. DL can be supervised, unsupervised, or reinforcement learning. This depends on the expected outcome and output one desires to achieve. With the tremendous amount of available data, DL can extract information better than conventional machine learning algorithms. There are four categories based on the primary method in DL approaches, which are convolutional neural networks (CNNs), restricted Boltzmann machines (RBMs), autoencoder, and sparse coding [48]. Figure 7 indicates the basic structure of the DL algorithm.

DL algorithms learn by tuning the weight (w) and bias parameters (b) of the network. This tuning process is performed by evaluating the prediction and the expected output. The algorithm evaluates the prediction quality through a loss function, for example, the mean square error function, after inputs are passed to its outputs. The adjustment of weight and bias parameters is made by a process called backpropagation, which employs the gradient descent method. The *w* and *b* parameters’ updates are done in the opposite direction to the loss function. After updating, the algorithm repeats the computation of the loss function after another iteration of the prediction.

## 4. Machine Learning for Resource Allocation in Vehicular Networks

Authors have different objectives when designing a resource allocation strategy using machine learning. These objectives can be in the form of balancing resource utilization, fulfilling QoS or the quality of experience (QoE) of users, enhancing power transmission efficiency, minimizing delay, or maximizing the entire system’s weighted sum rate. Each of these objectives determines the design of the resource allocation strategy along with the parameters it will involve. This section discusses the role of machine learning in resource allocation for vehicular networks.

### 4.1. Supervised Learning

Supervised learning for vehicular network resource allocation has two important roles. First, the regression task in supervised learning is utilized to predict the resource allocation solution for a given dataset. Second, supervised learning is a tool for learning the dynamic environment of the system to be optimized. In the latter role, a supervised learning algorithm can be implemented to enhance the reinforcement learning algorithm’s allocation decision effectiveness.

#### 4.1.1. Regression for Resource Allocation Decision

Supervised learning needs a dataset as an input and target data when predicting resource allocation—as required by the system. This dataset can be obtained from a simulation tool such as mininet emulator [49] or derived from other simulations involving optimization algorithms [50,51,52,53]. The supervised learning algorithms utilized by previous authors that we surveyed are shown in Table 2. The dataset involved in the learning process has a strong correlation with the objective of the resource allocation designed. For example, the channel condition and power transmission level correlate with the vehicle’s position and mobility. The authors in [49,50,51,52,53] exploited this relation to build the strategy by making use of the observation dataset. From our literature study, we identify the correlation between sensing-based and location-based observations for resource prediction mechanisms.

Channel condition is commonly utilized as an input parameter in resource allocation strategy. Its parameters can be in the form of channel state indicator (CSI) or signal-to-noise ratio (SNR)/signal-to-interference-plus-noise ratio (SINR) values of the moving vehicles. The minimum SNR/SINR value correlates with the system’s lower QoS bound [50]. A central controller then utilizes reported SNR/SINR values to observe the system’s global condition. Then, it makes the allocation decision based on some pre-determined rules to obtain a global optimum. However, CSI utilization for resource allocation can increase the system’s overhead. Furthermore, an accurate CSI value is difficult to obtain due to the vehicles’ high mobility.

To overcome inaccurate CSI problems, supervised learning can be utilized to predict the CSI values of vehicles by observing their geographical positions. Using this method, the global condition can be observed while reducing the system’s overhead. Vehicles’ mobility, represented by speed and direction, can also be exploited to estimate the channel condition [56]. Vehicles’ movements are relatively stable during a period of time due to road conditions. The key strategy in this scheme is to observe the non-linear correlation between CSI/SNR and vehicles’ position to the central controller. Models that are often used to predict channel conditions are autoregressive (AR); band-limited process (BP); and sum-of-sinusoids (SOS) models—where the AR-based model has higher accuracy with lower complexity compared to the other two models [57]. Thus, the AR-based model is suitable for implementation under near-realistic channel conditions.

Moreover, supervised learning can also be applied to allocate optimum and efficient transmission power. Vehicle-to-RSU/BS distances, antenna sensitivity, and vehicle movement can be used as input parameters for the learning process [55,56]. For example, the authors in [55] implemented logistic regression to determine the power fraction which can flexibly decrease BS/RSU transmit power according to the vehicle–RSU distance. Additionally, in [54], DNN was utilized to determine the optimal transmit power according to the channel realization and the channel gain of V2I and V2V links. Although training the algorithm requires a longer amount of time, the algorithm can provide a fast solution for a dynamic resource allocation decision.

#### 4.1.2. Dynamic Environment Observation

Supervised learning can be applied to dynamic environment observation. This state environment will then be utilized by RL algorithms to create better policies for the system. Learning from raw data observed by vehicular environment needs longer computational time. Furthermore, safety applications often contain duplicate information. Implementing a neural network in environmental observation can help decrease the learning time to improve the policy decision-making process with a shorter time in the resource allocation mechanism. In addition, supervised learning can identify hidden patterns in a large dataset. Parameters such as vehicular trajectory, position, and resource availability in the system can also be predicted using supervised learning to help RL algorithms make the best policy decision. Table 3 presents the supervised learning implementation for dynamic environmental monitoring in several papers that we surveyed.

A deep recurrent neural network (RNN) was applied in [61] to investigate the pattern of resource availability based on the vehicle-to-RSU positions. The authors utilized parked and slow-moving vehicles in the coverage area to alleviate the RSU traffic burden. This method gave better performance than heuristic and other machine learning resource allocation algorithms. Hou et al. [58] made use of the relation between the social graph and communication graph to select resources among nodes. The social graph was widely used in Internet data analysis to reflect trust between users. It became the basis of the offloading scheme for the video processing task. Hou et al. also implemented long short-term memory (LSTM) algorithm to predict the vehicles’ trajectory in order to select serving RSU in the simulation area in [59]. Then, this information was further fed to the QL algorithm to help decide the best resource allocation policy for the nodes.

### 4.2. Unsupervised Learning

The role of unsupervised learning in resource allocation strategy is to provide a cooperative method for a group of vehicles with similar characteristics to improve the resource allocation procedure [62]. A central controller such as BS/RSU can select intermediate vehicles or a cluster head (CH) to support the data dissemination process. Using the cooperative method, scheduling overhead and data collision probability can be reduced [63]. In addition, applying the clustering method can improve the vehicular network’s stability and scalability [64]. Clustering is a technique to group vehicles with similar characteristics such as geographical vicinity, traffic pattern, the application’s QoS, and interference value. Clustering can be implemented in network routing, tracking, congestion estimation, and resource allocation [46,65]. This subsection only discusses the application of clustering in resource allocation mechanisms mainly implemented in vehicular networks, as studied in several papers.

#### 4.2.1. Cluster Formation Strategy

When grouping vehicles, a cluster analysis method needs input parameters to decide which nodes have similarities and dissimilarities. There are many methods unsupervised learning algorithms to group data points. The main difference is in how the similarity of data points is defined. In addition, it can also be determined by variable distribution and cluster shape. Cluster formation can be performed in a centralized or distributed manner. In the distributed clustering scheme, each node obtains the surrounding nodes’ information and tries to find a group of vehicles with similar or almost similar characteristics. In this scheme, CH is chosen as a cluster coordinator based on specific stability parameters. BS/RSU acts as a controller for centralized clustering, which gathers information with some criteria for all nodes in its coverage area. Then, these nodes are grouped according to their similarity values.

Clustering for the resource allocation mechanism generally uses a centralized scheme where BS/RSU groups vehicles to improve the resource algorithm’s effectiveness. In [66], the coverage area was divided into clusters. In each cluster, the federated DRL on a small timescale was implemented to obtain a robust global model. For each of the new V2V pairs activated, this global model can be implemented to reduce the learning time. This algorithm performs better than the conventional decentralized learning scheme. A similar approach was also carried out by [67] where CH was chosen as QL agents according to its stability, average speed, link quality, and neighborhood degree. The CH selection in each cluster to support a BS/RSU in forwarding the packet was also performed by [64,68]. Using CH support, the efficiency and effectiveness of resource allocation can be improved.

#### 4.2.2. Clustering Model

In our study, we classified the clustering models implemented for cooperative resource allocation in vehicular networks according to the clustering paradigm described in [69]. Table 4 depicts the clustering strategies and its similarity parameters used in our surveyed papers.

In correlation clustering [68,70,71,72], the number of clusters that can be established in the system depends on the similarity data points. This clustering method tries to find a harmonious partitioning, where disagreement between data points is minimized, whereas the agreement is maximized [73]. Data points in correlation clustering can be viewed as connecting graphs with a classifier function *f*. Spectral clustering, which also has its roots in graph theory, uses the similarity graph *G* for data representation. Instead of using pairwise similarity or pairwise distance, such as correlation clustering, spectral clustering abstracts data points based on the eigenvector from the adjacent matrix, such as the Laplacian matrix from the dataset or constructed graph. The authors in [66,74,75] implemented spectral clustering with interference and location-based similarity to group vehicles in an area. Readers that are interested in how spectral clustering is built to separate data points can refer to [76].

Partitional clustering, such as by the *K*-means algorithm, precisely allocates a data point in a cluster. It needs the information of how many *k* clusters it has to build in order to separate data points. This clustering model iteratively relocates a data point until the optimal partition is achieved. Wang et al. [80] created a self-adaptive clustering model for mobile vehicles to efficiently distribute the bandwidth between DSRC and LTE users. This clustering method utilized the iterative self-organizing data analysis technique (ISODATA) formula to enable the dynamic cluster formation according to the vehicles’ environment changing. In contrast to partitional clustering, a hierarchical model determines the number of clusters according to the dendrogram it creates in every step. The dendrogram represents the similarity and order of the clusters. Cao et al. [78] implemented Ward’s linkage clustering algorithm with the sum of the square errors’ calculation to merge two similar clusters to obtain an optimal cluster with maximal similarity.

### 4.3. Reinforcement Learning

Generally, the process of creating a system model and the learning phase between many papers on RL implementation for vehicular resource allocation have similar characteristics. RL has four main components, which are the state, agent, action, and reward. This system can implement a centralized or decentralized learning model to construct the best policy for the resource allocation strategy. This learning model influences the agent selection of the system. An agent observes the state space and takes action that can maximize its long-term reward. Action is the resource allocation decision, such as a resource block (RB) selection, power transmit ratio allocation, offloading decision, spectrum ratio, etc. The reward has strong correlation with the design objective. It can be in the form of the combination of average delay in the transmission process, the system’s cost, resource utilization, the system’s capacity, and QoS or QoE satisfaction. In this section, we discuss two main differences, which are the learning method and state parameterizing strategies of the RL approach between the works of the literature surveyed.

#### 4.3.1. Learning Method

RL for resource allocation can be performed in a centralized or decentralized manner. In centralized learning, a central controller such as a BS/RSU or a CH can be an agent to perform the learning process based on the system’s current state. On the other hand, decentralized learning uses V2V links or V2V pairs as agents to perform the learning process and autonomously make decisions. Centralized learning has advantages in eliminating packet collision compared to the decentralized scheme. Moreover, it has more stable links due to the BS/RSU transmission support and promotes more services with different QoS requirements. However, centralized learning has higher complexity and signaling overhead for the scheduling process. Decentralized learning can achieve sub-optimum resource allocation with lower complexity and learning time. Nevertheless, decentralized resource allocation suffers from the hidden node problem that affects the packet delivery ratio of the system compared to the centralized scheme [81]. In our studied literature, more than 75% of resource allocation mechanisms were performed in a centralized manner.

Decentralized learning involves multiple agents in the learning process. Agents observe their local environment and select the optimal action to maximize their rewards. In comparison, centralized learning can use single or multiple agents in its learning process. A single agent in a centralized scheme, which is a BS/RSU or a CH, collects the environmental information through beacons sent by vehicles in its coverage area. The system can make a globally optimal strategy based on this information. Centralized multi-agent learning can be seen in [82], where a central controller manages several RSUs in allocating resources in its coverage area. The resource allocation strategy was made at each time slot. The author assumed that RSUs had similar and stable environments. An updating policy was carried out by implementing the soft actor critic (SAC) in the central controller as an off-policy RL algorithm. This updated value was sent to each edge agent so that agents could adjust their resource allocation strategies accordingly. The simulation results proved that the proposed algorithm could significantly increase the system’s performance compared to other schemes.

#### 4.3.2. State-Based Allocation Strategy

RL implementation in vehicular networks has a dynamic system state. This state changes with time. The state is a set of parameters describing a system. The set of parameters in a state representation must be factors that can influence the learning result so that the agent can learn successfully. This set of parameters has a relation with the resource allocation’s objective. It also depends on the system model. For example, references [83,84] have different approaches to solve the resource allocation problems, although both resource types are similar. The authors in [83] tried to allocate resources for V2V pairs by using available V2I links. The model they built was closely related to the influence of interference to nearby V2I and V2V links. On the other hand, reference [84] designed an energy-efficient vehicular network which employed the vehicle–RSU distances to determine the transmit power and resource allocations. Due to these differences, we classify the resource allocation strategy using RL in the vehicular network into three categories, namely (i) location-based; (ii) sensing-based; and (iii) availability-based strategies.

These classifications are based on the state space parameters which are used to observe the environment. The combination of (i) and (ii) can happen when a decentralized learning scheme is implemented. This is because the V2V link uses the position and interference parameters to determine its best allocation strategy. The combination of (iii) with (i) or (ii) happens when the vehicular network uses SDN-based or the Fog/Edge computing framework. The combination of all these strategies occurs when a mobile agent such as a UAV is involved [85] or vehicles with rich or unexploited resources can provide it to its neighboring vehicles [82]. This mobile agent uses the location-based strategy to observe its surrounding nodes, the sensing-based strategy to prevent interference between nodes, and the availability-based strategy to determine the amount of resources allocated according to the surrounding nodes’ requests. Table 5 shows each of our surveyed literature strategies in modeling RL for vehicular network resource allocation.

The location-based strategy uses state parameters such as vehicle–RSU distance, vehicle trajectory, vehicle speed, and vehicle density. In a centralized learning scheme, an agent obtains information on the number of nodes with their positions and/or mobilities in its coverage area. Then, the agent makes a resource allocation strategy based on state changes during each period [84,86]. Atallah and Assi [87] utilized the weighting technique for each vehicle and chose vehicles with the nearest locations to save energy. Xia et al. [62] and Arkian et al. [67] divided the coverage area according to the vehicles’ locations and mobilities, and then selected a CH for each area as an agent. RL was utilized to select an auxiliary vehicle that can help forward packets in a cluster.

The sensing-based strategy is mainly applied in the decentralized scheme with V2V links as agents in its learning process. State parameters for each V2V pair can be a combination of local channel information, interference value from the neighboring transmitter, and traffic loads. In direct communication, the V2V link suffers from limited spectrum resources. In order to increase the amount of allocated resources, the interference values from surrounding nodes can be essential parameters that can influence the agents’ decision-making process. For example, in [88], V2V links as agents chose their spectrum and transmitted power with minimum interference for their V2I and V2V links in the surrounding area. The authors divided transmitted power into three levels which agents chose according to its state. A cooperative scenario was created by [89], where coexisting agents that generally compete with each other were made to collaborate to increase the system’s fairness. In this work, an agent has a weight according to its traffic condition. The asymmetric Nash bargaining solution was utilized as the cooperative method with a DRL approach to achieve convergence.

The availability-based strategy uses parameters such as resource availability and the number of resource requests required by the system. Resource availability can be in the form of a resource state and its distribution, the number of the available BS/RSU and/or the vehicles with unutilized resources. This strategy is usually implemented in the SDN-based vehicular network, vehicular Cloud network, and vehicular fog/edge computing resource allocations. He et al. [22] proposed a connected vehicles framework that can separate network resources. These separated resources can be gathered as a pool of resources for several different vehicular applications. The authors utilized a software-defined and virtualized vehicular network, which is managed by a central controller. The agent must choose an available BS/RSU that could provide resources for each vehicle that made a resource request. Liu et al. [97] divided BS’s resource into several slices to serve various requests from V2X. Each slice is a deep deterministic policy gradient (DDPG)-agent that attempts to serve resource requests from its users. These agents tried to meet the minimum requirement of users while maximizing the sum-utility of their resources.

### 4.4. Deep Learning

DL consists of neural network layers that are useful to approximate a solution for an optimization problem. It is capable of creating a new feature from datasets without predetermined information. DL is adaptable and can be implemented in supervised, unsupervised, or reinforcement learning. This section describes the role of DL implemented in other machine learning categories that we found in our literate study.

Generally, DL is used to enhance the machine learning algorithm’s performance during resource allocation management. DL implementation in supervised learning treats the optimization problem as a black box [13] and more accurately extracts the relation between parameters. It is also proven that by using DL, the non-linear relation between parameters can be extracted to provide better resource allocation decisions with negligible overhead [52]. Two machine learning models were implemented in IoV by [51]. The first model predicts the resources needed at the edge side, while the second utilizes RNN to predict future utilization. RNN is a robust DL algorithm with internal memory that is suitable to perform resource prediction. By predicting resource availability, the system’s effectiveness is guaranteed by avoiding over-provisioning. J. Gao et al. [54] implemented a DNN to approximate the weighted minimum square error (WMMSE) value by learning the mapping between the channel power gain as the input and the optimal power allocation as output in the V2V and V2I links. The results indicate that implementing DNN supervised learning improved the system performance compared to conventional supervised learning.

The emergence of DNN in computer vision also drew attention to its implementation to improve other machine learning algorithms. While reinforcement learning is proven to solve complex objective problems, it takes a long time to achieve the best policy. Furthermore, the curse of dimensionality makes it unsuitable for implementation in large-scale networks. DNN as a function of approximation is implemented in reinforcement learning to train the learning process. Applying DNN in reinforcement learning shortens the learning process and improves reinforcement learning performance.

DNN improves reinforcement learning performance by advancing the learning process to make policy in an uncertain environment. X. Chen et al. [91] implemented the DRL algorithm to develop a resource management strategy. Since several resources and two applications were involved, conventional Q learning was not possible due to the high number of actions in the current state. The DL algorithm was used to predict the reward value for several different actions in their system. Compared to other conventional reinforcement learning algorithms, DRL converges faster. In [93], DNN was used as part of DQL to approximate the reward function, where multiple parallel DNNs were applied to generate the computational decision. The simulation results show that the convergence process linearly increases with the number of DNNs involved in the system.

## 5. Challenges and Opportunities of Machine Learning in Vehicular Network Resource Allocation

The implementation of machine learning offers a low complexity solution for complex resource allocation problems in vehicular networks. Furthermore, it can dynamically adjust its solution according to the changing environment of the vehicles. Nevertheless, this implementation can be challenging due to some conditions, such as changing topology due to vehicle mobility and various applications with different QoS requirements. This section mainly discusses the challenges of the machine learning-based resource allocation strategy in vehicular networks.

### 5.1. Environment Modeling

Real-life experiments involving vehicular nodes and BS/RSU are difficult to perform. For this reason, researchers implement their ideas in simulations. Creating environment models for vehicular network simulations requires various parameters and criteria. In vehicular networks, many nodes are mobile. Thus, choosing the simulation and network topology such as node distribution, propagation model, and mobility model, which reflects real-life traffic and network conditions will lead to higher complexity. Some assumptions are made to simplify this process, which can unfortunately reduce the mobility characteristics of vehicles in the simulation.

The neural network can be a powerful tool to extract patterns from a large dataset. It can distinguish the hidden pattern and label it at a fast rate. Liang et al. [91] exploited it by implementing neural-network-based RL for the dynamic demands of resources in a mobile environment. The authors utilized a planning algorithm to map the action values for specific state–action pairs. This mapping was applied as the initial sample of the neural network. Then, RL was implemented to update parameters and train the neural network. This mechanism can eliminate the need for strong pre-set assumptions when building the model. Furthermore, inaccurate model estimation problems can be avoided so that optimal results can be achieved.

### 5.2. QoS Guarantee

The vehicular network offers safety and non-safety applications to improve traffic safety and comfort. These applications, such as active safety applications, traffic management, and infotainments, have different QoS requirements which need to be fulfilled. The objective of a resource allocation strategy is to ensure that nodes have enough resources to satisfy the QoS requirements of the applications involved in the transmission process. However, satisfying all the QoS requirements of various applications can be challenging due to some applications’ conflicting requirements, such as high data rates with negligible latency. Some authors, for example, in [22,58,90], preferred to focus their works on designing resource allocations for a specific type of service due to its importance in the vehicular network environment. This method can reduce the complexity of the algorithm used in resource allocation. However, reliable V2X communication needs a resource allocation scheme that can satisfy users’ various applications.

Tayyaba et al. [49] designed a flow-based resource allocation framework in the SDN-based virtualization for the vehicular network under 5G. This uses a traffic classifier that can divide traffic flows into three classes: priority queue, bandwidth sensitive queue, and no strict queue, according to each flow sensitivity and delay boundary. A central controller dynamically allocates bandwidth according to the applications’ QoS requirements. Incoming packets create queues according to their priority, and the controller assigns resources according to its length. A stochastic process was used to simulate the framework and create datasets multiple times. Then, these datasets were fed to the DL block so that the system could learn and predict the resource allocation strategy for the incoming traffic. In [87], an energy-efficient adaptive resource allocation to facilitate different traffic types was created. The system was built on the assumption of an energy-limited RSU. The state explorations gathered by the agent were the network and traffic conditions to determine the amount of service request loads and the number of vehicles residing in the coverage area. The QL algorithm was utilized as the optimal scheduling policy to dynamically allocate energy consumption while achieving an acceptable level of QoS for its service requests.

### 5.3. Task Diversity

The machine learning tasks involved in a vehicular resource allocation mechanism can be separated into resource prediction, environment modeling, clustering for cooperative resource allocation, and a Markovian-based resource allocation decision. There are many machine learning algorithms that can fulfill each of these tasks’ objectives. Obtaining an optimum result requires a correct choice of machine learning algorithm and the parameters it uses in the system’s design. For example, in resource prediction, the dataset is in the form of time series. A supervised learning algorithm combined with DL such as LSTM can be a good choice since the previous time step is stored in the memory. It can predict the resources required by V2V and V2I links more accurately than CNN and DNN algorithms. However, it needs a slightly longer time to allocate the resources compared to the other methods [49].

Neural network-based algorithms for dynamic environment modeling were used in some scenarios [59,60]. The neural network was widely used in pattern recognition for its ability to perform complex identification in a short amount of time. Another task is grouping nodes into several smaller clusters with similar attributes. We can see that many works were done using graph-theory-based clustering in our literature study. Using graph-theory, the inner structure of the dataset can be more clearly investigated. For Markovian-based resource decisions, an agent or agents need to create the best policy that can maximize long-term rewards while optimizing system performance. DRL has the ability to handle high-dimensional action space and states. It has a self-improvement capability to select the best action. Resource allocation in vehicular networks is a model-free-based RL problem. It is important to understand the resource allocation problem and abstract the task into several sub-problems, and investigate whether using machine learning can solve it. By understanding the problem, one can find a suitable machine learning algorithm to solve it efficiently and optimally.

### 5.4. Distributed Approach

In our surveyed literature, the majority of resource allocation strategies were performed by the central agent. It makes allocation decisions after collecting various state information from its environment. Although the central learning strategy was proven to have better performance than the decentralized scheme, the increase in nodes and applications involved in vehicular networks will increase the complexity of the centralized algorithm. This will affect the computational burden of the central agent when gathering global environment information from surrounding nodes. Therefore, the decentralized learning approach, where nodes can make local observations and autonomous decisions, can be a less complex solution. Furthermore, it is preferable to implement distributed resource allocation with decentralized learning when the number of nodes in the system is large.

For example, Zhang et al. [66] formulated a joint optimization problem to enable mode selection for V2V links in a cellular-based V2X. The objective is to maximize the V2I capacity while meeting the V2V link’s requirements. Using decentralized learning, where each V2V link is an agent, a two-timescale federated DRL was created. Vehicles in the same cluster cooperated in training the DRL model, and the global model could be applied to the newly joining V2V links. The result showed that the algorithm could outperform the decentralized method while achieving competitive results with the centralized method.

## 6. Conclusions

This paper presents a survey of machine learning algorithms implemented in vehicular network resource allocation. We mainly focused on the role of machine learning in the resource allocation strategy. We present how each machine learning category is utilized to provide a dynamic resource allocation scheme. Based on the parameters involved in resource selection, we classified resource allocation strategies into the sensing-based, position-based, and availability-based ones. These strategies correlate with the resource allocation objective and the type of resource involved in the mechanism. Although AI implementation is promising in enhancing vehicular network performance, some challenges also need to be considered when designing the solution. Finally, this survey paper can help readers understand the role of machine learning algorithms and their strategy in vehicular network resource allocation mechanisms.

## Figures and Tables

**Table 1:** Existing survey papers and their contributions and gaps.

Reference #	Theme	Contribution	Gap
[6]	Vehicular resource allocation based on interface types	Presenting various resource allocation methods based on interface types of the vehicular network	Deeper analysis for machine learning implementation was not discussed
[10]	Vehicular Cloud resource management	Identifying and examining the resource management tasks that were carried out in the vehicular Cloud	Discussing the resource allocation strategy for C-V2X only in the Cloud infrastructure
[11]	Application of deep reinforcement learning (DRL) for communication and networking	Investigating the application of DRL from the literature review and delivering the tutorial of DRL to address issues in communication and networking	The strategy to utilize the DRL algorithm in vehicular networks—especially for resource allocation was not yet discussed
[12]	Overview of machine learning algorithms and applications in vehicular networks	Explaining the application of machine learning categories in wireless networks	The further strategy of utilizing machine learning algorithms to manage resource allocation in vehicular networks was not shown
[13]	Deep learning in wireless resource allocation	Focusing on the effect of deep learning implementation in wireless resource management	Only deep learning is discussed

**Table 2:** Supervised learning algorithms to predict resource requirement.

Reference #	Dataset Parameter	Algorithm
[49]	QoS flow types	CNN, DNN, LSTM
[50]	SINR	ANN
[51]	Traffic flow	RNN
[52]	CSI and position	DNN
[53]	Mobility and resource request	SVM
[54]	Power	DNN
[55]	Power	Logistic regression

**Table 3:** Supervised learning for environmental observation.

Reference #	Dataset Parameter	Algorithm
[58]	Shareable resource and routing path for packet delivery	Graph theory (social graph and communication graph)
[59]	Vehicle mobility	Unspecified
[60]	Number of vehicles	DNN
[61]	Resource availability	D-RNN

**Table 4:** Clustering algorithms and similarity parameters utilized to enable a cooperative resource allocation mechanism.

Reference #	Similarity Parameter	Algorithm	Clustering Paradigm
[62]	Mobility	Stability-based clustering	Other
[66]	Channel gain	Spectral clustering	Spectral
[67]	Position and mobility	Unspecified	Other
[68]	Interference between platoon	Vertex coloring	Correlation
[70]	Mutual interference	Graph clustering	Correlation
[71]	Interference	Graph partitioning	Correlation
[72]	Position and velocity	Graph partitioning	Correlation
[74]	Position	Spectral clustering	Spectral
[75]	V2V link quality	Spectral clustering	Spectral
[77]	Task delay and size	KNN	Partitional
[78]	Position	Ward’s linkage	Hierarchical
[79]	Position (for safety applications) and CSI (for infotainment applications)	Gaussian similarity and distance-based clustering	Partitional
[80]	Position and velocity	K-means with ISODATA	Partitional

**Table 5:** Reinforcement learning strategy for resource allocation.

Reference #	Objective	Algorithm	Learning Method	Strategy Based on		
				Sensing	Availability	Position
[22]	Vehicular network resources orchestration	DRL	Centralized		✓	
[58]	Offloading for mobile video apps on the basis of social graphs	Continuous time MDP	Centralized		✓	
[59]	Maximizing the successful content fetching for non-safety application in a mobile vehicular node	QL	Centralized		✓	
[60]	Resource allocation for out-of-coverage V2V communication	DRL	Centralized		✓	✓
[61]	Efficient vehicular fog computing resource allocation to minimize service latency	RL	Centralized		✓	✓
[62]	Increasing traffic efficiency and capacity by implementing a cooperative scheduling mechanism	RL	Centralized			✓
[66]	DRL-based decentralized resource allocation for C-V2X links to increase system capacity and satisfy QoS	DRL	Decentralized	✓		✓
[67]	Efficient, stable, and reliable Cloud resource management in the vehicular Cloud architecture	QL	Centralized			✓
[77]	Intelligent resource allocation and task offloading to improve next generation vehicular network	RL	Centralized	✓		
[82]	Resource allocation for high mobility vehicles to satisfy applications’ QoS requirements	DRL	Centralized	✓	✓	✓
[83]	Resource allocation for V2X communication underlying cellular networks	DDPG	Decentralized	✓		
[84]	Joint energy efficient scheduling and routing framework for delay tolerant application	QL	Decentralized			✓
[85]	Flight resource allocation to minimize packet loss ratio during nodes’ data transmissions	DDPG	Centralized	✓	✓	✓
[86]	Joint resource allocation for delay-sensitive application in MEC-based vehicular network	DRL	Centralized			✓
[87]	Downlink resource allocation with power and QoS constraints	QL	Centralized			✓
[88]	Unicast and broadcast resource allocation	DRL	Decentralized	✓	✓	
[89]	Resource sharing in SDN-based heterogeneous vehicular network	DRL	Decentralized	✓		
[90]	Resource allocation to minimize the Perception reaction time (PRT) for safety and non-safety applications	DRL	Centralized		✓	
[91]	Vehicular Cloud resource allocation to maximize the QoS and QoE	RL	Centralized		✓	
[92]	Communication and computation offloading to alleviate RSU’s burden	Distributed DQL	Centralized		✓	
[93]	Resource provisioning to balance resource utilization and QoS satisfaction in virtualized network for V2V links	DRL	Centralized		✓	
[94]	Efficient joint resource management for UAV	DDPG	Centralized		✓	✓
[95]	Resource management for V2V link broadcast application with minimum latency and interference	DRL	Decentralized	✓		✓
[96]	Optimizing system performance by implementing an intelligent offloading mechanism in vehicular edge computing	DRL	Centralized	✓		
[97]	QoS-based resource allocation	DRL	Centralized		✓	
[98]	Dynamic resource allocation for SDN-based and virtualized vehicular network to satisfy QoS requirements	A3C	Centralized		✓	
[99]	MEC-based vehicular network offloading to reduce energy while satisfying applications QoS constraints	DQN	Centralized		✓	✓
[100]	Enabling the dynamic orchestration of resource allocation to improve system performance	DRL	Centralized		✓	
[101]	Queue priority resource allocation in 5G vehicular network	DQL	Centralized		✓	
[102]	Vehicular Cloud resource allocation where vehicles allocate or request resources from the resource pool	AVARAC (semi-MDP based)	Centralized		✓	
[103]	Distributed spectrum sharing mechanism where V2V links reuse the pre-occupied spectrum of the V2I link	DRL	Centralized	✓		
